# Six different extremely calcified lesions of the brain: brain stones

**DOI:** 10.1186/s40064-016-3621-3

**Published:** 2016-11-09

**Authors:** Yurdal Gezercan, Vedat Acik, Gökhan Çavuş, Ali Ihsan Ökten, Emre Bilgin, Hakan Millet, Burak Olmaz

**Affiliations:** Department of Neurosurgery, Adana Numune Training and Research Hospital, Serinevler Mah, Ege Bağatur Bulvarı, 01260 Yüreğir, Adana, Turkey

**Keywords:** Aneurysm, Angiomatous meningioma, Benign mesenchymal neoplasm, Cerebral calculi, Hamartoma, Pituitary stone

## Abstract

**Background:**

This study aimed to extend clinical documentation of cerebral calculi by reporting six cases of cerebral calculi with distinct etiologies and localizations.

**Methods:**

We evaluated the age, sex distribution, presenting symptoms, neurological examination findings, pathology results, and location of the calcifications of six patients with intracranial calcifications.

**Results:**

Three of the six patients with brain stones were female (50%), and three were male (50%). The patient ages ranged from 12 to 46 years. A radiological examination of each patient’s cranium was performed with pre-operative cranial computed tomography and magnetic resonance imaging. All of the lesions were completely excised. The patients’ pathologies were determined to be distinct hyalinization, dystrophic calcification, hamartoma, ossification developing from widespread pituitary adenoma tissue, benign mesenchymal neoplasia, a mass consisting of sporadically ossified fibrous tissue, and angiomatous meningioma with distinct hyalinization and fibrosis.

**Conclusions:**

Intracranial calcifications are a common phenomenon in neurosurgical practice. However, brain stones, as well as solid calcifications also termed cerebral calculi, are rarely encountered. Brain stones can be classified on the basis of their etiology and localization. Additionally, we suggest that lesions smaller than 1 cm might be classified as calcifications and those greater than 1 cm as brain stones. We further suggest that the differentiation between calcification and brain stones might be based on size. These pathologies typically manifest as seizures and are occasionally identified during routine brain tomography. Meningiomas constitute an important portion of extra-axial calcifications, whereas tumorous and vascular causes are more prevalent among intra-axial calcifications.

## Background

Brain stones, or cerebral calculi, are large solitary or multiple intracranial calcifications (Tiberin and Beller 1963). Although intracranial calcifications are observed frequently, brain stones are less frequently encountered. Brain stones typically manifest as seizures but can also be detected incidentally during brain tomography. They can be classified on the basis of their localizations and etiologies. Extra-axial stones can be physiological or tumor related. Intra-axial stones can be caused by neoplastic [oligodendroglioma, medulloblastoma, primitive neuroendocrine tumors (PNETs), dysembryoplastic neuroendocrine tumors (DNETs), and ganglioglioma], vascular [cavernous malformations, arteriovenous malformations (AVMs), and chronic vasculitis], infectious [TORCH, cytomegalovirus (CMV), herpes simplex virus (HSV), and parasites], congenital [Sturge–Weber syndrome and neurofibromatosis (NF)], endocrine, or metabolic (Fahr’s syndrome and hyperparathyroidism) factors (Celzo et al. 2013). Tumorous and vascular factors are prevalent causes of intra-axial stones, whereas meningiomas constitute an important portion of extra-axial stones (Celzo et al. 2013). The deterioration of calcium regulation is the primary reason for stone development. Neovascularization and intralesional arteriovenous shunt formation during the development of many tumors increase the blood supply to the tumorous tissue, and these conditions result in hemorrhaging that leads to necrosis within the tumorous tissue. Intracellular calcium regulation deteriorates as a result of this necrosis, and this process leads to calcium deposition (Osborn et al. 2004; Knaut et al. 2013). When vascular factors are prevalent, calcium regulation deteriorates in association with chronic venous ischemia. Intracellular calcium build-up increases, thus resulting in dystrophic calcification (Celzo et al. 2013).

After brain stones are identified, the treatment is typically surgical. However, the etiology and localization must be considered when planning the surgery, and the patient must be prepared accordingly.

## Methods

We evaluated the age, sex distribution, presenting symptoms, neurological examination findings, pathology results, and location of the calcifications of six patients with intracranial calcifications.

## Results

### Case 1

**Fig. 1:**
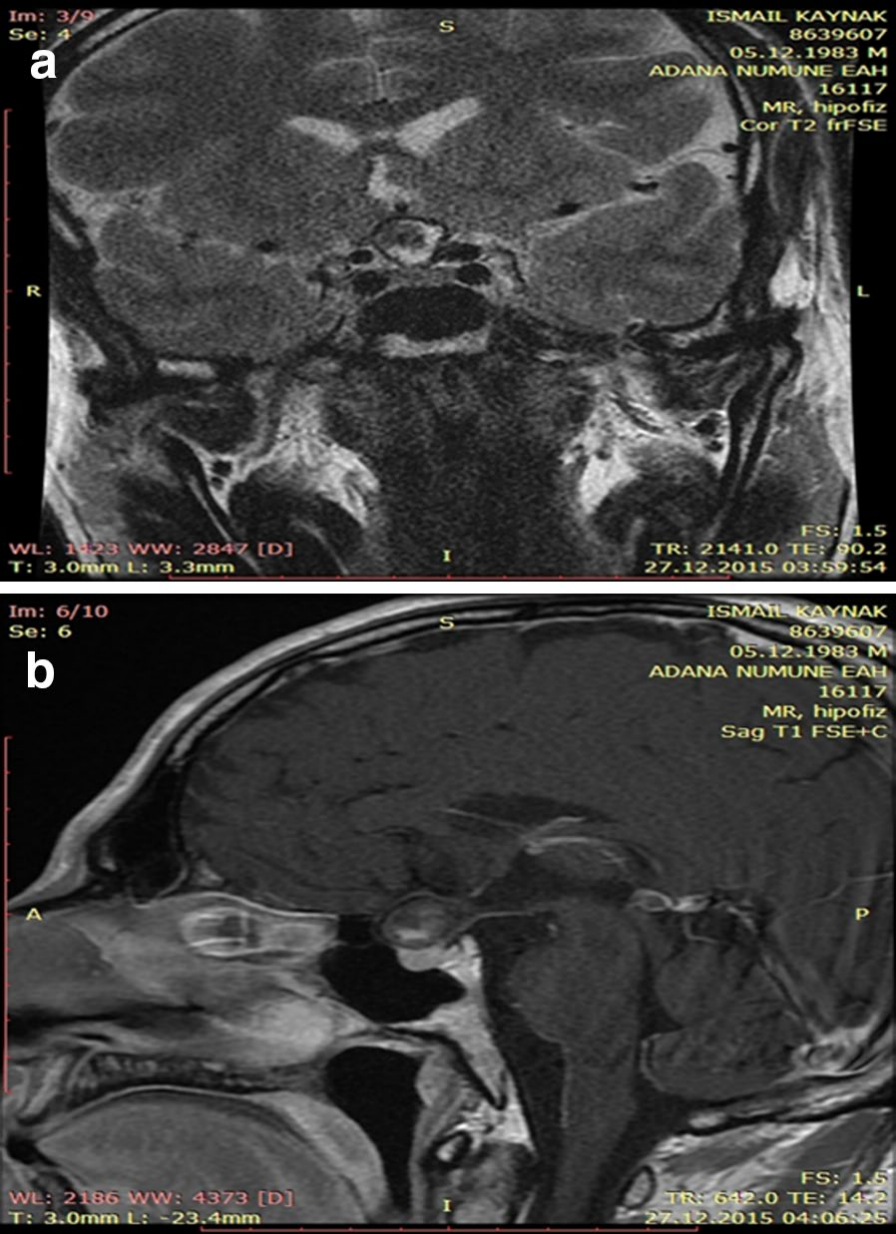
**a**, **b** Contrast-enhanced T2A and T1A images of the patient

**Fig. 2:**
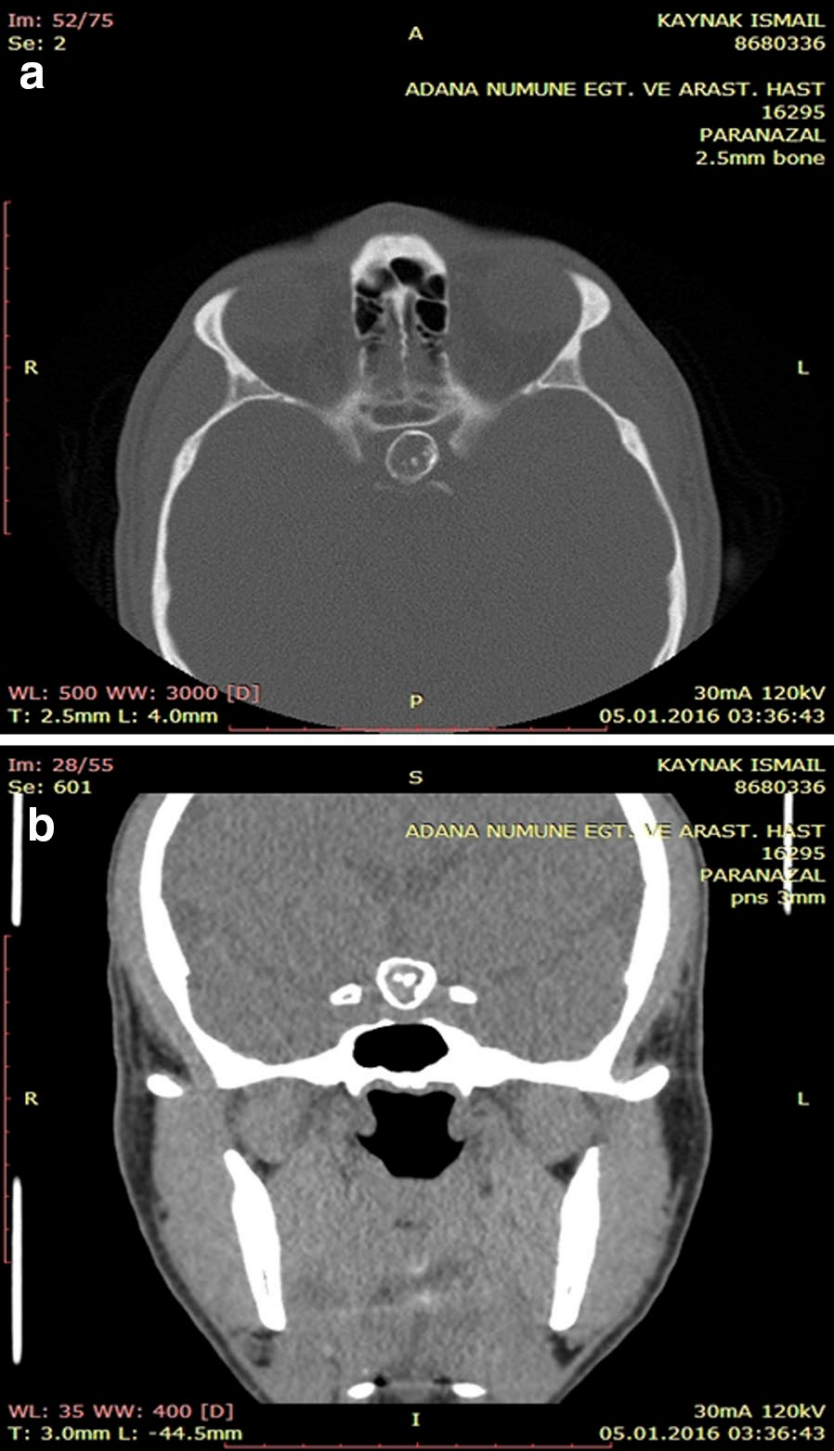
**a**, **b** The lesion was observed on the axial and coronal sections of a preoperative paranasal sinus CT of the patient

**Fig. 3:**
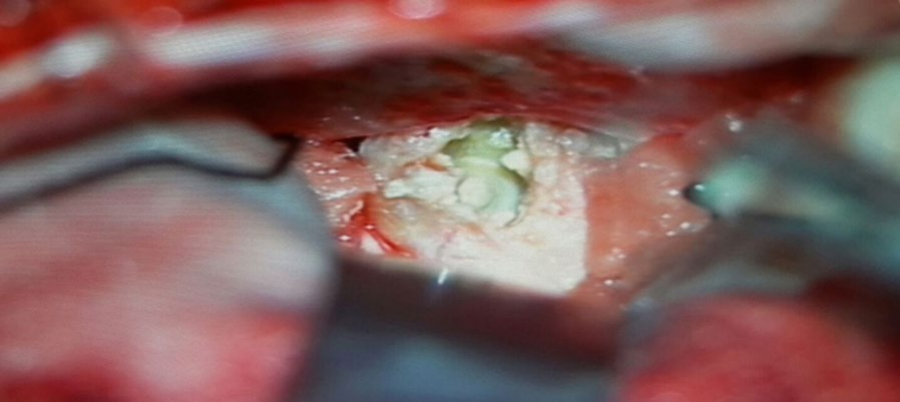
The lesion upon dissection. The connection of the aneurism with the basilar artery and the vortex formed by the blood circulation are clearly detectable

A 32-year-old male patient was admitted with complaints of headache and blurred vision. MR images of the patient revealed a non-contrasting, well-circumscribed lesion lodged in the pituitary gland; this lesion was intense and sporadically hypointense in T1A imaging and heterogenically hyperintense in T2A sections (Fig. 1a, b). Paranasal CT scans revealed a well-circumscribed ossified mass in the same area (Fig. 2a, b). A clinical examination of the patient’s visual field and vision revealed bitemporal hemianopsia. No pathology was detected through cerebral angiography. The patient underwent surgery, and a right pterional craniotomy was performed. The lesion was accessed via Sylvian dissection. Cutting into the lesion was abortive; therefore, a high-speed drill was utilized to open a hole, which was enlarged with forceps. A hollow aneurysm had a vascular connection, and a vortex formed by the blood was clearly visible after the lesion was opened (Fig. 3). When the lesion was carefully lifted up from the side, its relationship with the basilar crest was observed. Subtotal removal of the ossified formation entailed utilizing the high-speed drill to reduce the thickness of the lesion and utilizing forceps and alligator forceps to remove the lesion. An aneurysm clip was not used because there was no blood flow within the patient’s aneurysm. The patient had no additional neurological deficits in the postoperative period. The patient’s pathology was determined as distinct hyalinization and dystrophic calcification.

### Case 2

**Fig. 4:**
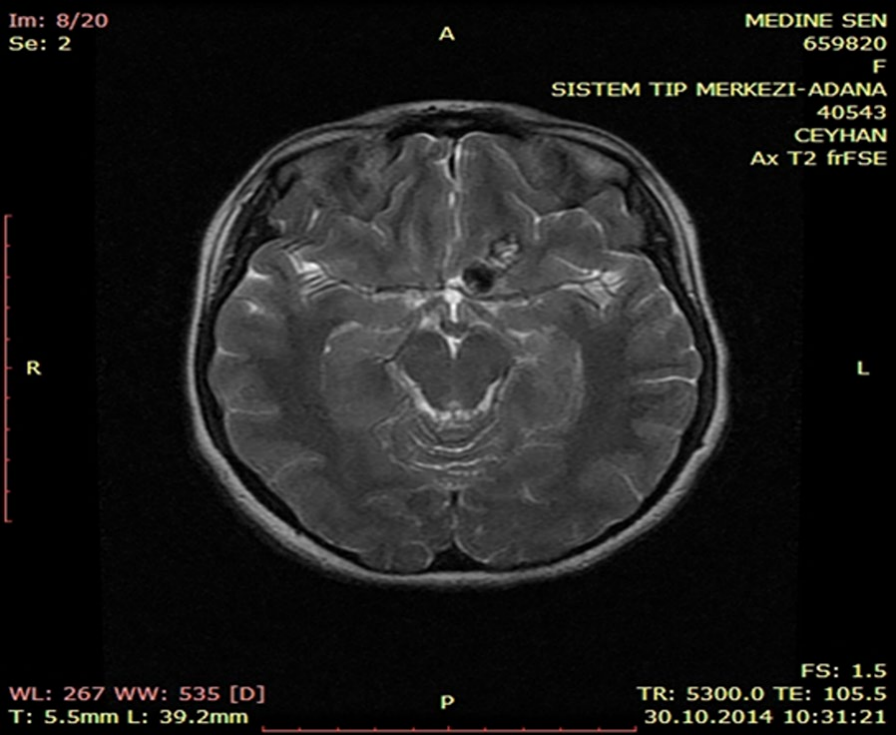
Axial T2A view of the formation

**Fig. 5:**
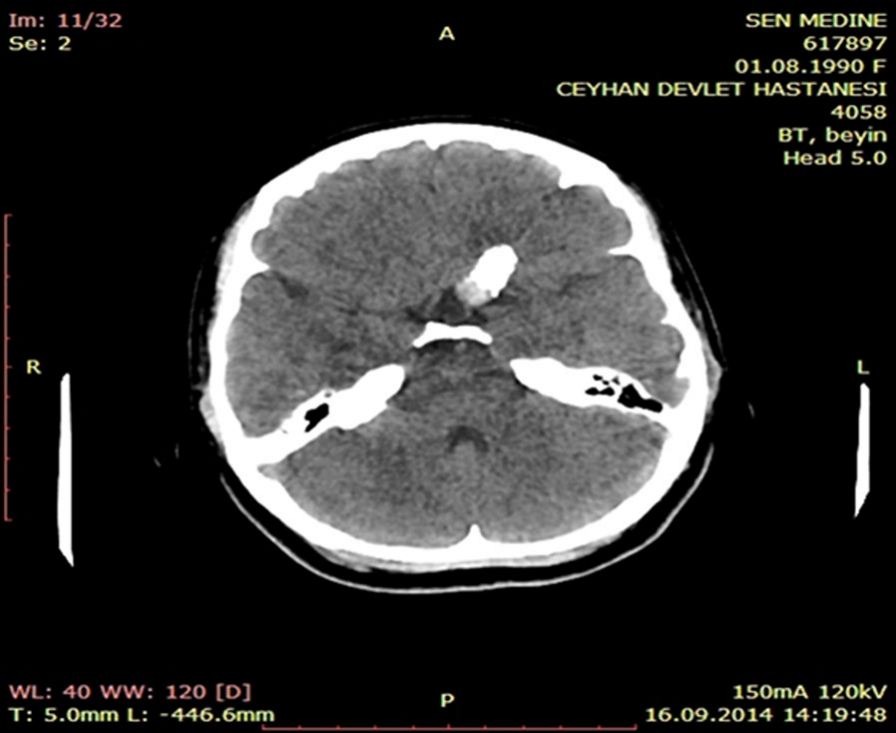
Cranial CT view of the formation

**Fig. 6:**
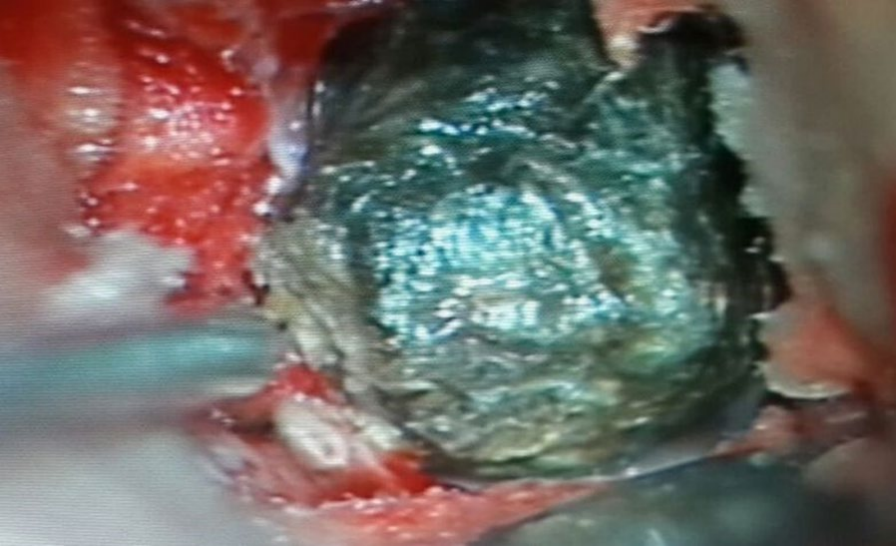
Formation as it was accessed via Sylvian dissection

**Fig. 7:**
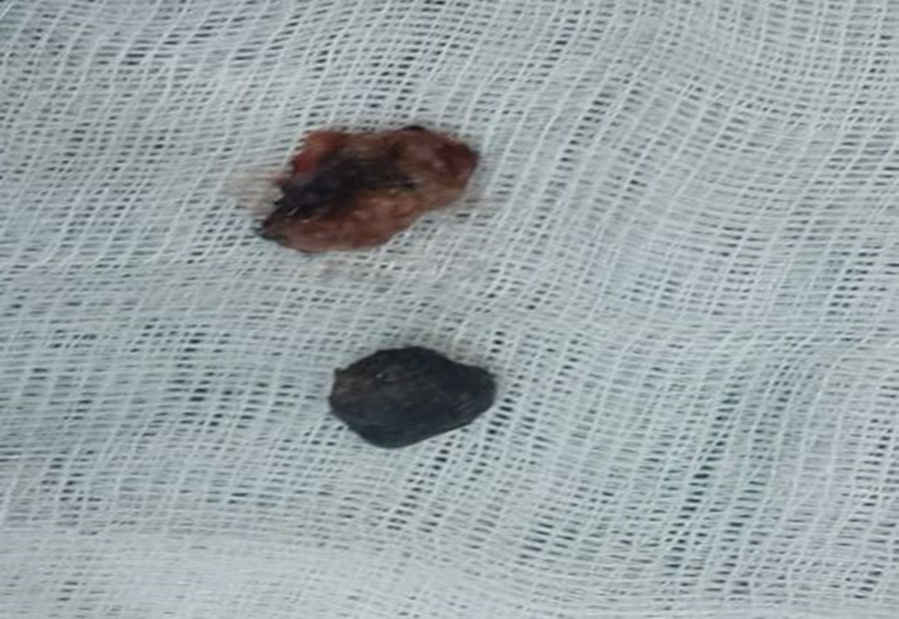
The lesion and the surrounding calcified tissue after removal

A 25-year-old female patient was admitted with complaints of headaches and behavioral changes. Cranial MRI of the patient revealed a non-contrasting lesion with irregular boundaries and hypointensity in T2A sections stretching along the sphenoid wing on the left frontal lobe (Fig. 4). Cranial CT of the patient revealed a mostly ossified formation with irregular boundaries that was hyperintense compared with the brain parenchyma located along the left sphenoid wing (Fig. 5). Pathology was not observed in MRI angiography of the patient. Left pterional craniotomy and Sylvian dissection were used to access the lesion. A hard cube-like black formation was observed (Fig. 6). The mass was dissected from its surroundings and was easily removed. Hard calcified lesions adjacent to the mass were completely excised (Fig. 7). Normal parenchymal tissue was reached. The patient’s pathology was reported as a hamartoma.

### Case 3

**Fig. 8:**
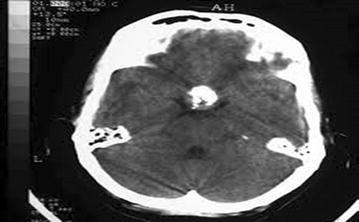
Unenhanced BT section showing ossification within a mass located in the sellar and suprasellar cisterns

**Fig. 9:**
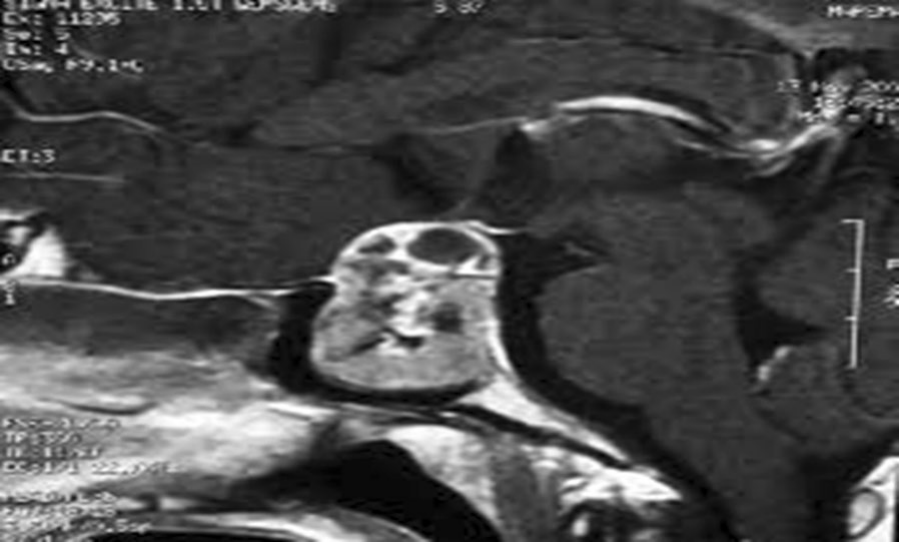
Enhanced sagittal T1 MR image of the patient. Sporadically hypointense areas indicative of calcification are detectable

**Fig. 10:**
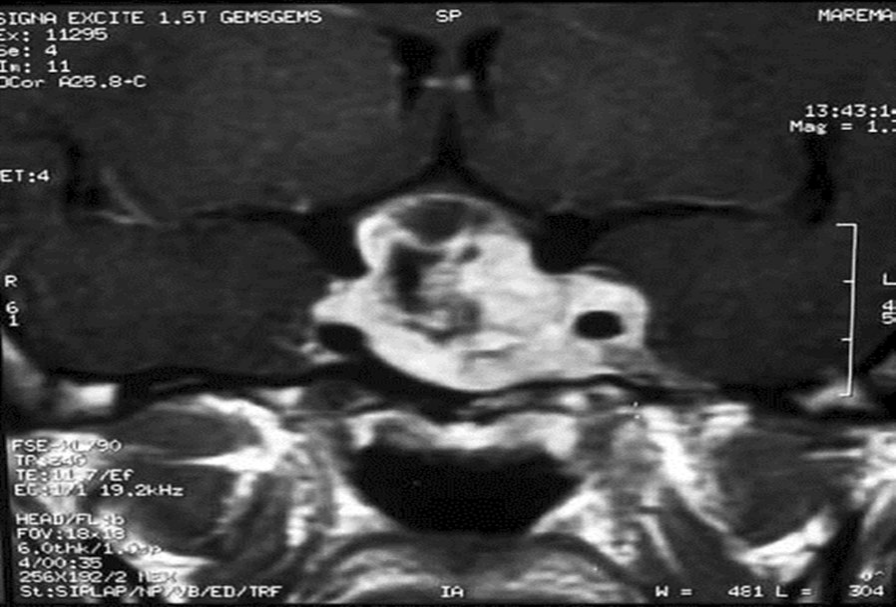
Contrast-enhanced coronal T1A image. Erosion of the sellar floor by the lesion is observed, and the mass extends into the sphenoid sinus, infiltrates into the left cavernous sinuses, compresses the optic chiasm, and surrounds the carotid artery

A 42-year-old male patient was admitted with complaints of increasingly severe headaches over the previous 6 months as well as deteriorated and blurred vision. Neurological examination revealed a bitemporal hemianopsia. Fundus examination revealed optical paleness. Amorphous calcifications with the density of bone, located within irregular boundaries and in a sellar and suprasellar location, were particularly interesting (Fig. 8). A heterogeneous mass filling the sellar and suprasellar cisterns and expanding to the sphenoid sinus while eroding the sellar base was detected in contrast-enhanced T1-weighted sagittal and coronal MR images (Figs. 9, 10). Only the prolactin levels of the patient were observed to be high (158.5 ng/ml) on the basis of hormonal examinations. The patient underwent operation via a pterional approach. A calcified mass in the pituitary adenoma was removed in pieces with forceps and alligator forceps. Calcified stones with chronic hemorrhaging, which were surrounded by necrotic adenoma tissue, were detected in the histopathologic examination. The patient’s prolactin levels returned to normal in the postoperative follow-ups. The patient’s pathology was reported as ossification developing from widespread pituitary adenoma tissue.

### Case 4

**Fig. 11:**
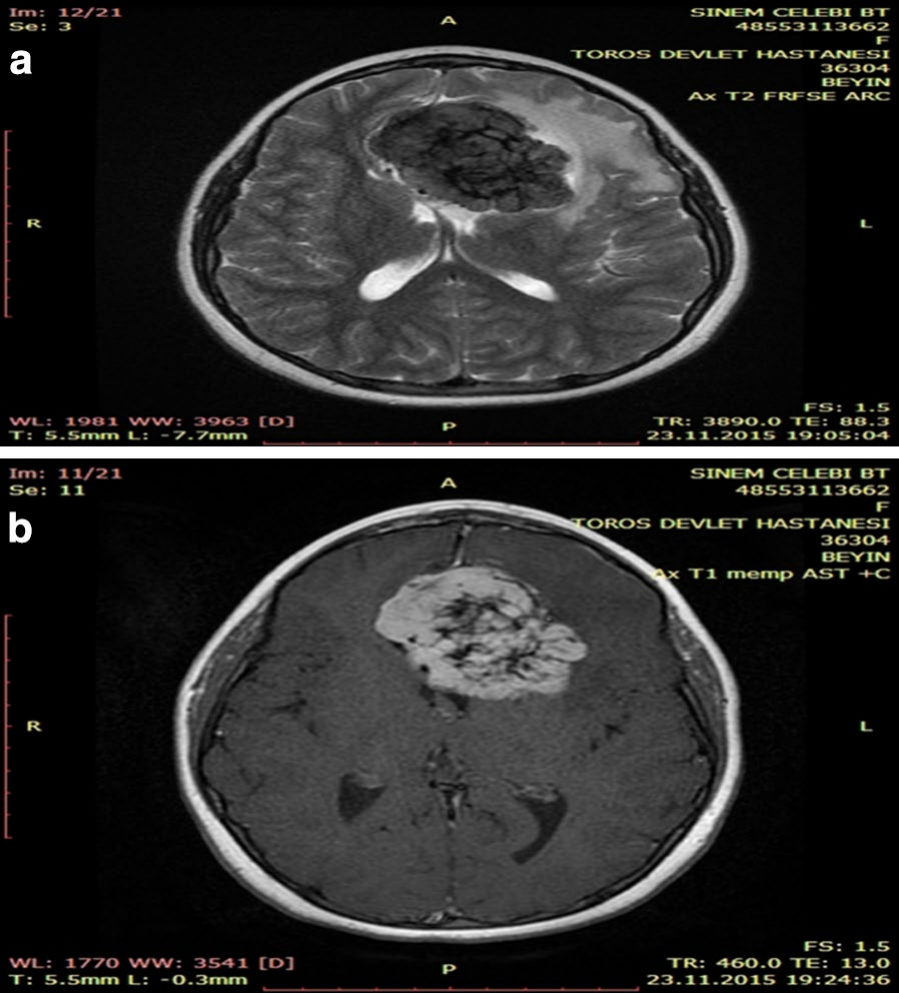
**a**, **b** Contrast-enhanced T2A and T1A MR images of the patient. Contrast uptake into the lesions is clearly detectable

**Fig. 12:**
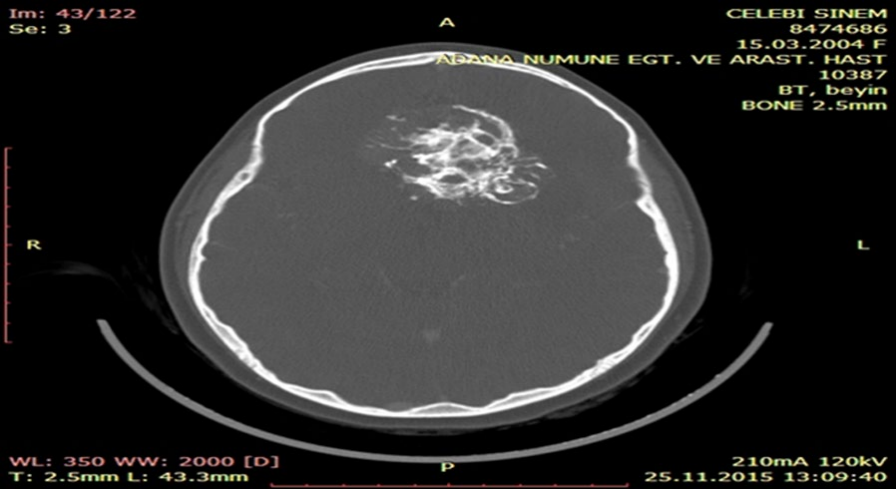
Calcification is clearly detectable in the bone window of the preoperative brain CT image of the patient

A 12-year-old girl was admitted with complaints of headaches and seizures. Cranial MRI of the patient showed an interhemispheric contrast-enhanced lesion in T1A and T2A sections appearing to be hypointense; this lesion extended upward from the base of the frontal lobe. Edema formed around the mass, and both frontal horns were compressed (Fig. 11a, b). Widespread regions of ossification were observed in the bone window of a brain CT image (Fig. 12). The tumorous tissue was accessed via an operation entailing left frontoparietal craniotomy. The tumor had a hard osseous structure and could not be aspirated. Excision of the tumorous tissue was initiated using forceps and a punch excision tool. At the base of the tumorous tissue, the tumor infiltrated into the left lateral ventricle but was not emanating from the ventricle. The lesion was removed as a gross total excision. In the pathological evaluation of the patient, areas of calcification and ossification, including cholesterol clefts with a collagenized appearance and certain areas showing local fibroblastic activity, were observed on a fibrous base. No atypia or pleomorphism was detected in the fibroblastic regions. Immunohistochemical assays of the epithelial membrane antigen (EMA), S100, vimentin, KI67, creatine kinase (CK), Leber congenital amaurosis (LCA), CD68, antibodies against glial fibrillary acidic protein (GFAP), BCL2, anaplastic lymphoma kinase (ALK), and synaptophysin levels were performed. Although the EMA results were negative, fibrous meningioma was not ruled out on the basis of the histomorphological findings. Staining assays to identify solitary fibrous tumors, inflammatory myofibroblastic tumors, schwannomas and histiocytic lesions produced negative results. The patient was diagnosed with benign mesenchymal neoplasia.

### Case 5

**Fig. 13:**
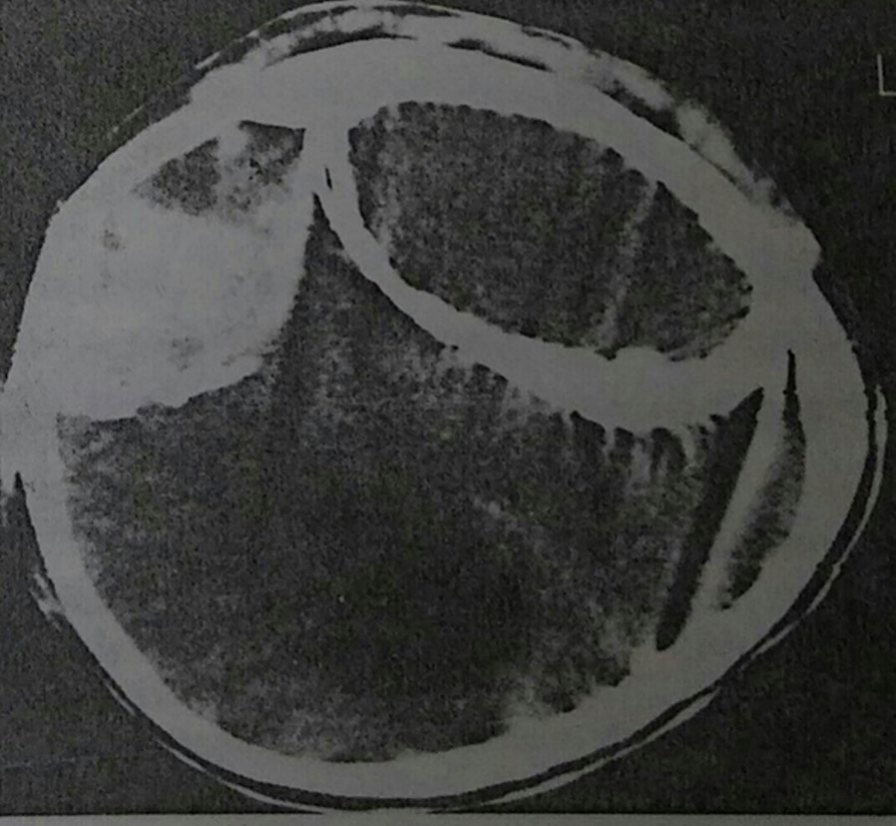
Preoperative BT image of the patient

**Fig. 14:**
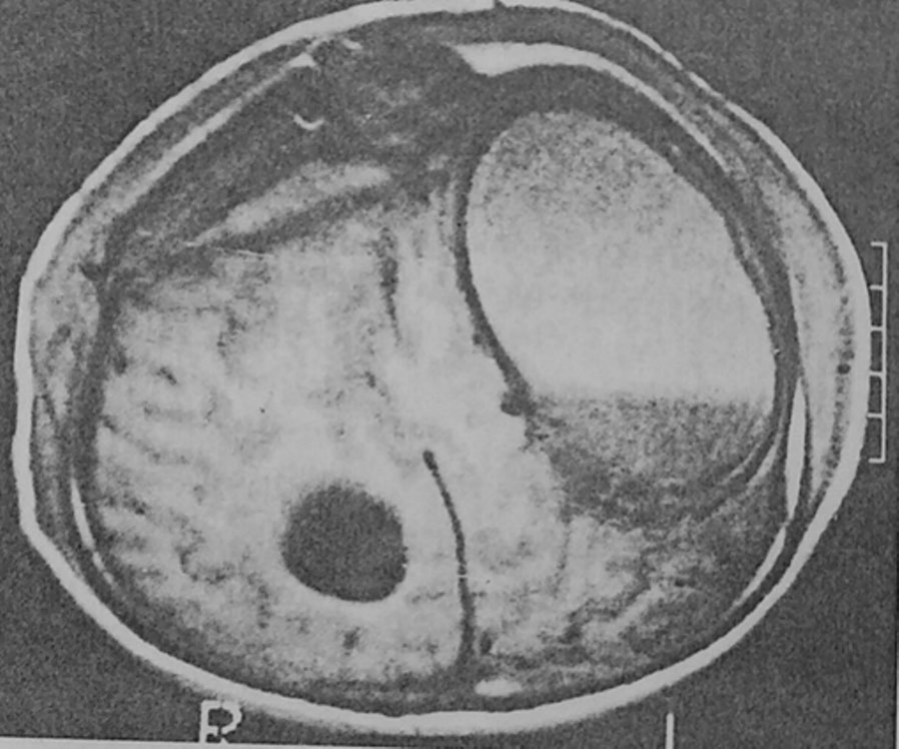
Cranial MRI of the patient prior to the 2nd operation

A 16-year-old male patient was admitted to the emergency department for head trauma from falling down a staircase during an epileptic seizure. His anamnesis revealed that he had been experiencing seizures for a long time. A neurological examination revealed unconsciousness, right dilated anisocoria, and a left-lateralized neurological deficit. Brain CT showed a right frontal epidural hematoma and a left liquid-filled calcified mass (Fig. 13). The patient underwent an urgent operation. The right frontal epidural hematoma was drained via a wide right craniotomy. No calcified mass was observed. The patient entered status epilepticus on the second postoperative day. Cranial MRI revealed that the calcified mass was exerting pressure and that the liquid content was undergoing changes in its characteristics, producing a shift effect (Fig. 14). Blood that was seeping into the lesion was thought to be the cause of the occurrence of differing densities. The patient underwent a second operation, and the ossified lesion was accessed via a wide left frontoparietal craniotomy. The mass was extremely hard and was completely removed using a 1–2-cm-thick rongeur. The liquid content of the ossified mass had a muddy texture. The membrane of the mass was fused with the cortex. Pathological analysis indicated that the mass consisted of sporadically ossified fibrous tissue.

### Case 6

**Fig. 15:**
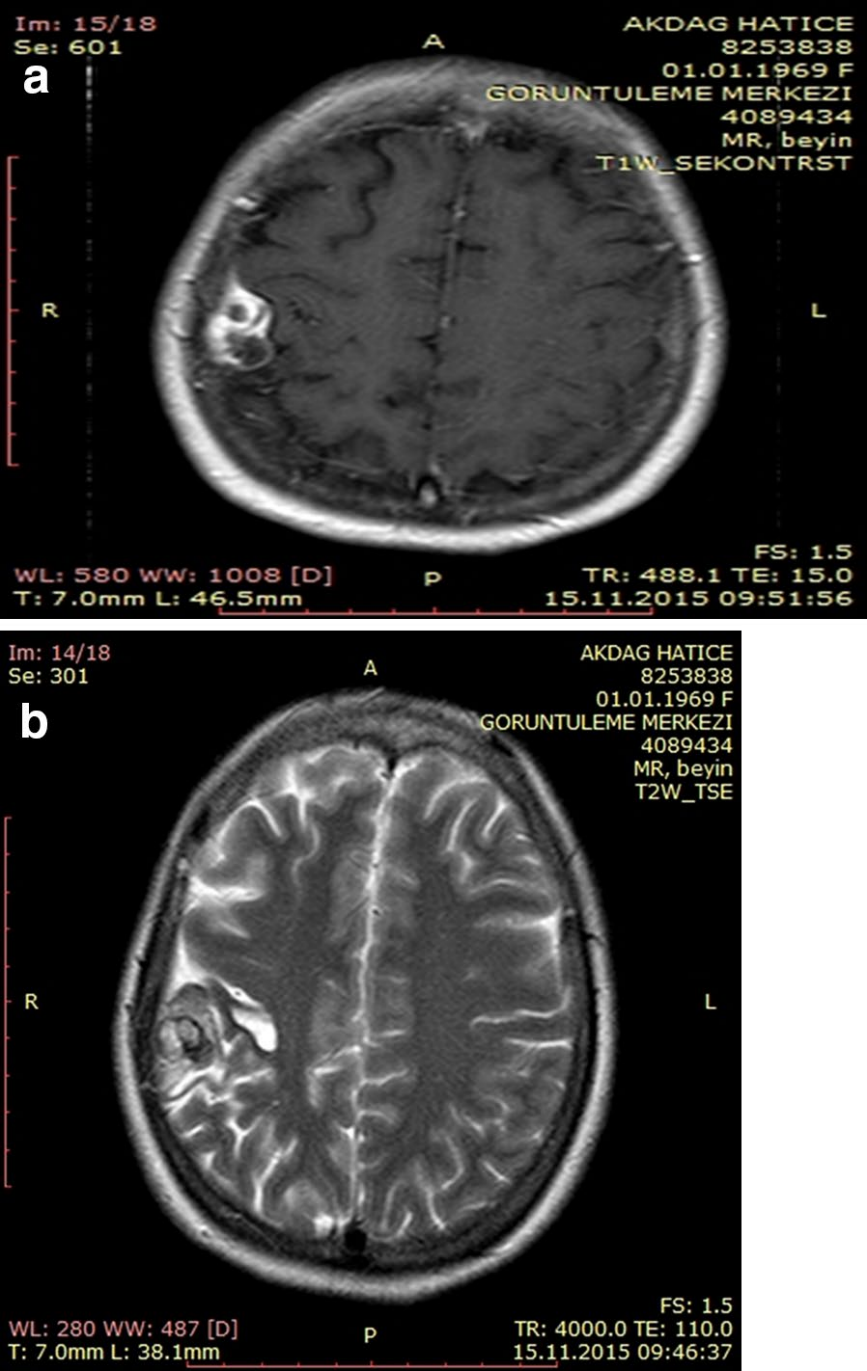
**a**, **b** Preoperative cranial MRI of the patient. T1A and T2A sections show a clearly contrast-enhanced hypo- and hyper-intense lesion at the right posterior parietal vertex

**Fig. 16:**
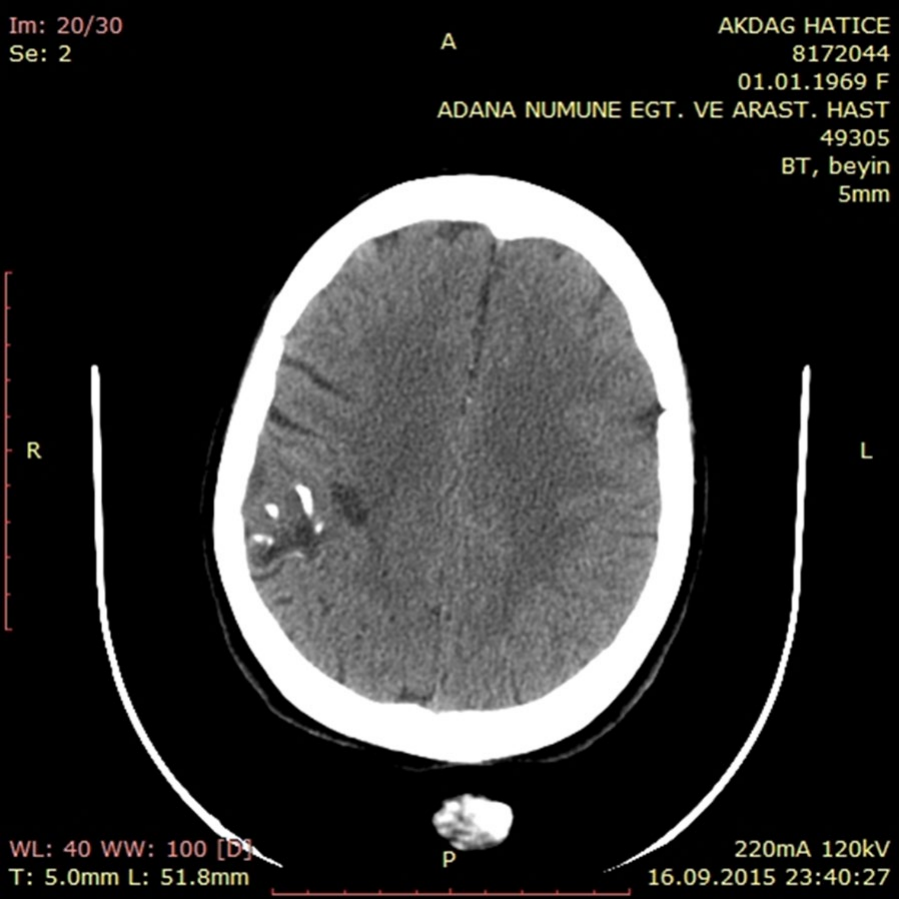
Preoperative cranial CT image of the patient

**Fig. 17:**
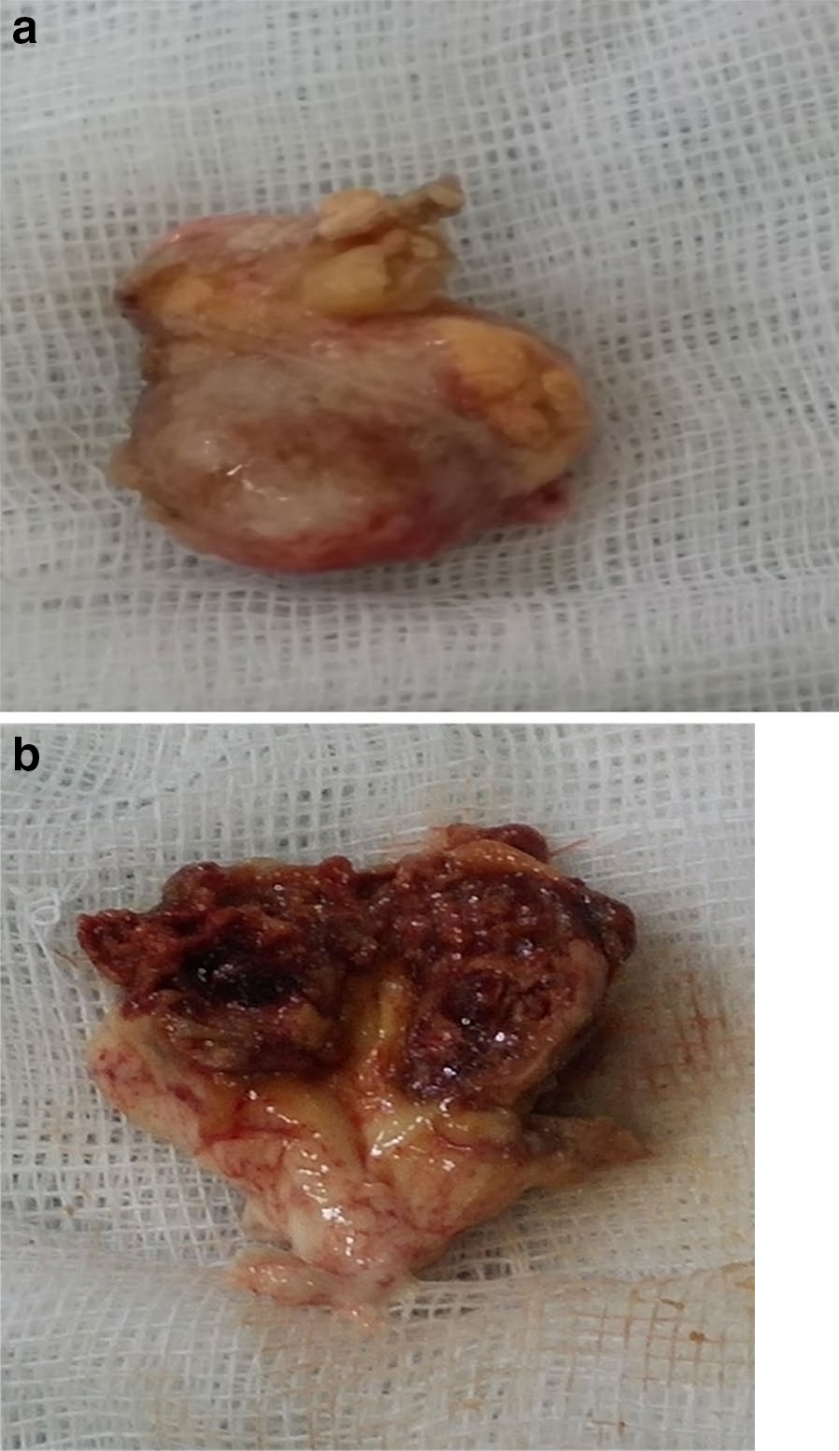
**a**, **b** Images of the tumor removed as two separate components. Vascular tissue is observed upon dissection of the tumor along the sagittal plane

A 46-year-old woman was admitted with a complaint of headache. Neurological examination of the patient produced normal results. Cranial MRI revealed a lesion that appeared to be predominantly hypointense and sporadically hyperintense in T1A images and predominantly hyperintense and sporadically hypointense contrast in T2A images (Fig. 15a, b). Cranial CT imaging of the patient revealed a slightly hyperintense mass with minimal calcifications in the cortical base periphery in the same location (Fig. 16). Cerebral angiography was planned for the patient, who appeared to have a calcified AVM. Angiography results of the patient were negative. The patient underwent surgery, and the lesion was accessed via a right posterior parietal craniotomy. The lesion appeared as hardly scrapable cemented material. The tumorous tissue was removed as a gross total dissection from the normal parenchymal tissue. A macroscopic inspection of the lesion after removal revealed a solid structure with a stone-like consistency. A soft mass of interior tissue was observed after the hard portion was penetrated. When the soft tissue was dissected, an interlaced vascular structure was clearly observed (Fig. 17a, b). The pathology of the patient was determined to be an angiomatous meningioma with distinct hyalinization and fibrosis.

## Discussion

Intracranial calcifications are frequently encountered pathologies. Large solitary or multiple intracranial calcifications are defined as brain stones or cerebral calculi. These pathologies occur much less frequently than intracranial calcifications. Brain stones can be located extra- or intra-axially. Extra-axial brain stones comprise tumors and exaggerated physiological calcifications. Intra-axial calcifications can be classified as vascular, neoplastic, congenital, infectious, and endocrine/metabolic etiologies.

Vascular causes represent a substantial portion of intra-axial lesions. The fundamental pathophysiological mechanism of the intra-axial lesion appearance is dystrophic calcification caused by chronic venous ischemia and hemorrhage. Brain stones caused by vascular factors were first defined by Shafey in a cavernous malformation (Shafey et al. 1966). Other vascular causes include chronic infarction, chronic vasculitis, AVM and aneurysm. Among vascular lesions, cavernous malformations are the lesions most frequently observed to be calcified, with an incidence rate of 40–60%. These lesions typically appear to be similar to popcorn kernels (Osborn et al. 2004). The second most frequently observed calcified vascular lesions are caused by AVM. Aneurysms appear much less frequently. One reason for this phenomenon may be that aneurysms become symptomatic and are treated. An observation of well-circumscribed lobe-shaped calcified formations in the suprasellar or parasellar region indicates possible aneurysms. Large aneurysms tend to calcify. Calcified material typically settles in the walls of the blood vessel, and such deposition is frequently related to intraluminal thrombosis (Pinto et al. 1979; Bhatia et al. 2011). In a study conducted on 22 giant aneurysms, spontaneous thrombosis has been observed in 50% of the aneurysms (Whittle et al. 1982). In our case 1, the lesion was a large basilar crest aneurysm located in the suprasellar cistern with a smooth circular shape. The vortex effect of the blood flow in the lumen of the aneurysm and its connection to the basilar crest were apparent after the lesion was cut open. We believe that the large aneurysm thrombosed over time and that the lumen turned to calculi via dystrophic calcification. No cases of a calcified basilar tip aneurysm were found in the literature.

Hemorrhage and necrosis are considered to be responsible for many instances of calcification in intracranial tumors. Hemorrhage is common in intracranial tumors, because the tumor essentially begins uncontrolled neovascularization to ensure its nourishment, forms intralesional arteriovenous shunts and consequently induces necrosis. This process deteriorates intracellular calcium regulation and leads to calcium build-up (Kasantikul et al. 1980; Trump et al. 1984). The most widely known calcified tumors are oligodendrogliomas, 90% of which display calcification (Makariou and Patsalides 2009). Additionally, 10–20% of medulloblastomas possess calcifications (Packer et al. 1999). However, metastases are rarely calcified (Drevelegas 2011). Meningioma calcification, with a density of 60%, has been observed in various studies (Makariou and Patsalides 2009; Kıroğlu et al. 2010). Calcification shows no correlation with tumor grade. However, calcification may be an indicator of successful treatment (Kalan and Burrows 1962). Many intracranial lesions display calcification. In the cases described in this manuscript, ossifying rather than calcifying pathologies were observed. Tuberous sclerosis manifests as hamartomatous lesions. The incidence rate of tuberous sclerosis in the most recent studies has been calculated as 1/10,000–20,000 (Bundey and Evans 1969). Tuberous sclerosis can appear in only cortical tubercles, and its occurrence is rare (DiPaolo and Zimmerman 1995; Yagishita and Arai 1999; Kang et al. 2007). Although these lesions are benign, they show contrast at a rate of 3–4% (Braffman et al. 1992). Tuberous sclerosis lesions appear to be hyperintense in T2A sections and iso- or hypo-intense in T1A sections (Nixon et al. 1989). Approximately 50% of these lesions appear in the frontal lobe (Houser and Gomez 1992). Other case series have reported calcifications in 50% of tuberous sclerosis cases (Menor et al. 1992). In brain CT scans, these calcifications appear as hypointense focal cortical or subcortical lesions. They have been reported to show gliosis, low levels of astrocytoma, atypical glial infiltration, micro-calcification spots and spongiform or vacuolar changes in myelin. In our case 2, the lesion was located in the frontal lobe, appeared hypointense in T1A and a mixture of hypo- and hyperintense in T2A sections and did not show contrast. In cranial CT, the lesion appeared as a mixture of hypo- and hyper-intensity. The patient had no additional clinical findings indicative of tuberous sclerosis. Our review of the patient’s radiologic and pathologic results indicated that the lesions would be the only form of tuberous sclerosis presenting with a cortical tubercle.

Pituitary adenomas and craniopharyngiomas are the most frequently encountered sellar–suprasellar masses. Although cyst formation and calcification in these masses are indicative of craniopharyngioma, calcification has been reported in pituitary adenomas, although rarely (Eldevik et al. 1996). The incidence of calcification in pituitary adenomas has been determined to be 0.2–0.8% radiologically and 5.4–25% histologically (Rilliet et al. 1981; Eldevik et al. 1996). Ossification is extremely rare. Calcification is frequently detected in prolactin-secreting adenomas (10–15%) but is less frequently detected in growth hormone-secreting adenomas (Shanklin 1948). Horiuchi et al. (1996) have identified a 42-year-old man with calcified pituitary adenomas. In a study conducted by Ke et al. (2010), a 21-year-old female patient was diagnosed with ossified prolactinoma, and a literature search revealed descriptions of 4 other patients with ossifying prolactinoma. Adenoma degeneration of various causes has been suggested to be the cause of pituitary calcification. Degenerative changes are frequently observed after pituitary apoplexy. Degeneration can also occur after medical treatments such as pituitary surgery, irradiation or bromocriptine administration (Shanklin 1948; Rilliet et al. 1981; Rasmussen et al. 1990). Degeneration has been suggested to cause proliferation in adenomas, thereby inducing osteoblastic and chondroplastic metaplasia in fibroblasts. It has been argued that ossification in the interstices of prolactinomas can be caused by the focal intensification of hyperprolactinemia in the systemic circulation or presence of autocrine prolactin within the tumor parenchyma (Ke et al. 2010). If limited by the sella, a lesion can be removed via the trans-sphenoidal route. However, in cases of suprasellar extensions, it has been reported that the transcranial route should be chosen because there may be adherence between the fibrous bands surrounding the ossified tissue and normal parenchyma (Webster et al. 1994). In our series, one patient harbored a prolactinoma. The transcranial route was chosen for the removal of the tumor because the lesion extended into the suprasellar region. The patient had not undergone bromocriptine therapy or radiotherapy, which might have caused degeneration. We believe that the pituitary adenoma began as ossification due to the autocrine effect of a high prolactin level as well as hemorrhage within the tumor.

Mesenchymal tumors stem from mesenchymal cells and are generally of benign character. These tumors can be found in a metastasized state or primary state; both forms are rare. Mesenchymal tumors can expand into the surrounding tissue and do not metastasize distantly. These tumors are typically located supratentorially; therefore, they can be confused with meningiomas. In our case 4, the mass expanded but did not undergo distant metastasis. The lesion could not be clearly distinguished from a fibrous meningioma despite immunohistochemical staining during the pathological assessment.

Although chronic subdural hematomas are frequently observed, their ossification is a rarely encountered phenomenon (Per et al. 2006; Moon et al. 2007). Although observed frequently in children and young people, chronic subdural hematomas can be found in any age group (Papanikolaou et al. 2008). Such patients may be asymptomatic or may have complaints of increased intracranial pressure, seizures, mental retardation or transtentorial herniation (Dammers et al. 2007). Calcified chronic subdural hematomas are typically late complications of head trauma (McLaurin and McLaurin 1966). These lesions can be observed as late complications in patients receiving shunts. Calcification is frequently observed alongside postmeningitic effusions (McKay et al. 1950). The mechanisms underlying the occurring calcifications are not completely clear. In chronic subdural hematomas, microscopic calcium deposits are observed on the membrane. Occasionally, these deposits could turn into calcifications or even ossifications. Several sources cite weak circulation and delayed reabsorption of blood in the subdural region as the cause of these lesions (Al Wohaibi et al. 2003). Parathyroid deficiencies provide a basis for calcification. Calcification generally begins in the outer portion of the capsule, and the membrane is thicker on the outside. MRI and brain CT evaluations of the patient before surgery can be helpful in determining the optimal treatment strategy for the patient. The management of calcified subdural hematoma is highly debated. Surgery is recommended if a patient has progressive neurologic deficits or increased intracranial pressure (Papanikolaou et al. 2008). In such patients, removal of the calcified material may not result in ensuring the control of calcification-related seizures. One of our cases was a young mentally retarded patient. He was admitted after a fall during a seizure. The calcified body was not removed during the first operation; instead, when neurological function was deteriorated, the calcified body was excised. Follow-up of the patient revealed no complaints of epileptic seizures.

Angiomatous meningiomas are a rarely occurring subgroup of meningiomas that are frequently observed in middle-aged women (Rao et al. 2008). Previous studies have reported a meningioma calcification rate of 2–3% (Cordera et al. 2002). Numerous vascular channels alongside a histopathologic appearance characteristic of a meningioma can aid in distinguishing angiomatous meningiomas from vascular malformations and hemangioblastomas (Kleihues and Cavenee 2000; Deb et al. 2010). Angiomatous meningiomas are categorized into the WHO grade 1 class. Perilesional edema caused by an increase in vascular structure is typically observed on MRI. These lesions appear to be hypointense in T1A sections but hyperintense in T2A sections and exhibit distinct contrast. These lesions are predominantly cortically located and manifest as seizures. In a histopathological study, Hasselblatt et al. have divided angiomatous meningiomas into micro and macro subgroups on the basis of the diameters of the vessels in the lesions (Hasselblatt et al. 2004). These lesions display a tendency to calcify, because their vascular content is high. Total excision is targeted in patients, but the relapse rate is very low in patients who undergo total lesion removal. In one of our cases, the patient was a woman. The lesion was cortically located, hypointense in T1A sections and hyperintense in T2A sections, with perilesional edema surrounding the lesion. Vascular regions were clearly detected upon macroscopic inspection of the lesion. Total removal of the tumor was achieved.

When we searched the literature, we found no report classifying these calcifications in terms of size. All of our patients’ lesions were larger than 1 cm. We recommend that lesions smaller than 1 cm be classified as calcifications and those larger than 1 cm be classified as brain stones.

## Conclusions

Unlike calcifications, brain stones are rarely observed pathologies. The basic pathophysiology of brain stones is the deterioration of calcium regulation resulting from necrosis, caused by frequently occurring hemorrhage. Importantly, the calcified lesions may be enriched in vascular tissue. These lesions may be detected in association with the occurrence of seizures or incidentally. The underlying pathology of calcification is typically benign, especially in tumors. The primary reason for this lack of malignancy might be that malignant tumors manifest symptoms at an earlier stage and are excised. Brain stones can have varying etiologies and localizations. We suggest that lesions smaller than 1 cm might be classified as calcifications and those greater than 1 cm as brain stones. We further suggest that the differentiation between calcification and brain stones might be based on size. Evaluations of the etiology, localization and size of calcifications must be conducted with great care when planning surgery on patients. Vascular pathologies must be considered, especially for round and well-circumscribed stones, and imaging techniques must be utilized for this purpose.

## Abbreviations

ALK: anaplastic lymphoma kinase
AVM: arteriovenous malformation
CK: creatine kinase
CMV: cytomegalovirus
CT: computed tomography
DNET: dysembryoplastic neuroendocrine tumor
EMA: epithelial membrane antigen
GFAB: antibodies to glial fibrillary acidic protein
MRI: magnetic resonance imaging
NF: neurofibromatosis
PNET: primitive neuroendocrine tumor
TORCH: toxoplasmosis, other agents, rubella, cytomegalovirus, herpes simplex
